# The complement system in intestinal inflammation and cancer

**DOI:** 10.1172/JCI188348

**Published:** 2025-10-01

**Authors:** Carsten Krieg, Silvia Guglietta

**Affiliations:** 1 Department of Pathology and Laboratory Medicine and; 2 Department of Regenerative Medicine & Cell Biology, Medical University of South Carolina, Charleston, South Carolina, USA.; 3 Hollings Cancer Center, Charleston, South Carolina, USA.

## Abstract

The complement system has emerged as a critical regulator of intestinal homeostasis, inflammation, and cancer. In this Review, we explore the multifaceted roles of complement in the gastrointestinal tract, highlighting its canonical and noncanonical functions across intestinal epithelial and immune cells. Under homeostatic conditions, intestinal cells produce complement that maintains barrier integrity and modulates local immune responses, but complement dysregulation contributes to intestinal inflammation and promotes colon cancer. We discuss recent clinical and preclinical studies to provide a cohesive overview of how complement-mediated modulation of immune and nonimmune cell functions can protect or exacerbate inflammation and colon cancer development. The complement system plays a dual role in the intestine, with certain components supporting tissue protection and repair and others exacerbating inflammation. Intriguingly, distinct complement pathways modulate colon cancer progression and response to therapy, with novel findings suggesting that the C3a/C3aR axis constrains early tumor development but may limit antitumor immunity. The recent discovery of intracellular complement activation and tissue-specific complement remains vastly underexplored in the context of intestinal inflammation and colon cancer. Collectively, complement functions are context- and cell-type-dependent, acting both as a shield and a sword in intestinal diseases. Future studies dissecting the temporal and spatial dynamics of complement are essential for leveraging its potential as a biomarker and therapeutic in colon cancer.

## Introduction

The intestinal tract is a highly specialized organ system responsible not only for nutrient absorption and waste excretion, but also for maintaining a delicate balance between host immunity and tolerance to the vast array of microbial and environmental stimuli it encounters. Central to this equilibrium is the integrity of the intestinal barrier, which relies on tightly regulated epithelial, immune, and microbial interactions. The complement system — traditionally known for its role in pathogen defense — has recently gained recognition for its critical functions in maintaining intestinal homeostasis and mediating responses to injury and infection. Beyond its classical extracellular roles, complement proteins are now understood to act locally and intracellularly, influencing a wide spectrum of physiological and pathological processes. This Review explores how dysregulation of the complement system contributes to intestinal inflammation and cancer, with a focus on emerging mechanisms, cell-specific functions, and potential therapeutic implications.

## Intestinal compartmentalization

**Figure 1:**
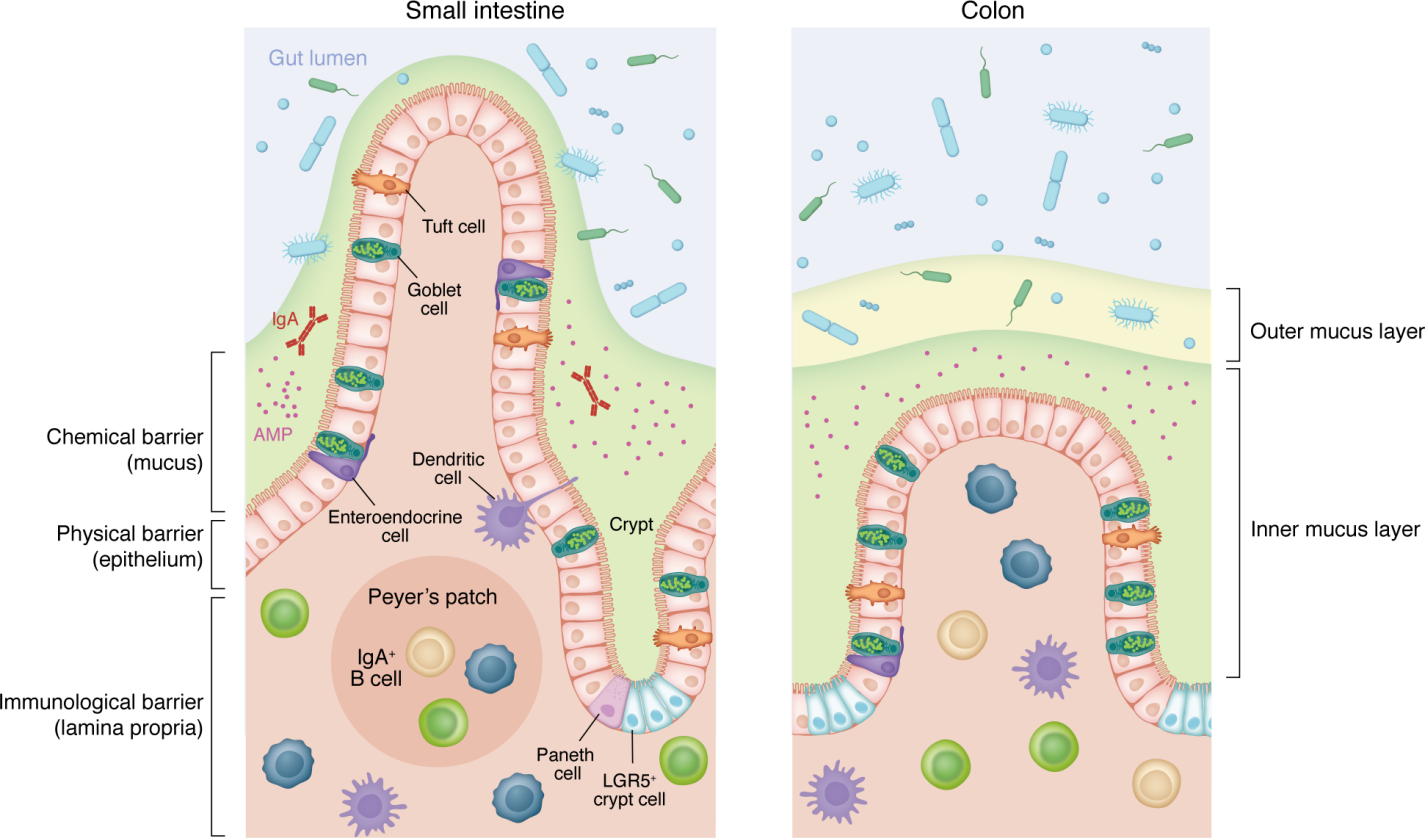
The composition of the intestinal barrier. The intestinal mucus creates a physical and chemical barrier that prevents direct contact between the luminal microbiota and the intestinal epithelium. Along the small intestine (SI), the mucus layer is relatively porous to different bacteria and thin. The colon (C) is equipped with a double mucus layer: the outer layer is relatively penetrable to bacteria; the inner layer is highly impenetrable to the microbiome (120–123). The intestinal epithelium comprises a single layer of heterogenous and highly specialized cell subtypes arising from common stem cell progenitors (Lgr5^+^ cells) at the bottom of the crypts. Intestinal epithelial cells (IECs) are tightly held together via strong cell adhesion proteins, forming a physical barrier that separates the contents of the intestinal lumen from the underlying lamina propria (124). Along with providing a physical barrier, cells within the intestinal epithelium produce and secrete molecules that help maintain intestinal homeostasis via a chemical barrier (124). Enterocytes (SI) and colonocytes (C) are the most abundant IECs. These cells recognize pathogen-associated molecular patterns via TLRs and NLRs (125–128). Enterocytes also express polymeric IgA receptors (pIgR) basolaterally, limiting intestinal permeability and susceptibility to intestinal inflammation and infections. Additional IEC types are goblet cells, which are responsible for the formation of the mucus layer through secretion of Muc2 mucin as well as other proteins; Paneth cells, which are found in the base of crypts within the SI that secrete antimicrobial peptides (AMP); tuft cells, which provide defense against helminth infections; and enteroendocrine cells, which secrete hormones. Intraepithelial lymphocytes (IELs) such as CD8^+^ T and γδ T cells are also found in the intestinal epithelium. Most immune cells are located in the lamina propria underneath the epithelial layer and in the gut-associated lymphoid tissues, which represent the key antigen sampling and adaptive immune inductive sites within the intestinal wall.

The intestine is equipped with a multilayer and multifunctional barrier composed by mucus, specialized intestinal epithelial cells (IECs) that provide a physical and chemical barrier, and immune cells (Figure 1). Alterations of virtually any of the single components of the intestinal barrier can result in the disruption of intestinal homeostasis and development of inflammation. Enhanced permeability promotes the translocation of luminal microbes across the barrier, ultimately initiating an inflammatory immune response involving production of inflammatory cytokines that further contribute to the disruption of the epithelial barrier and exacerbate intestinal inflammation, as extensively reviewed by Horowitz and collaborators (1). Moreover, as we discuss below, components of the complement system influence intestinal barrier integrity in complex ways. Breakdown of the intestinal barrier and subsequent loss of tolerance creates a cycle of inflammation that contributes to the pathogenesis of intestinal diseases such as inflammatory bowel disease (IBD), in which altered expression of various tight junction proteins has been observed (2–4).

## The complement system

**Figure 2:**
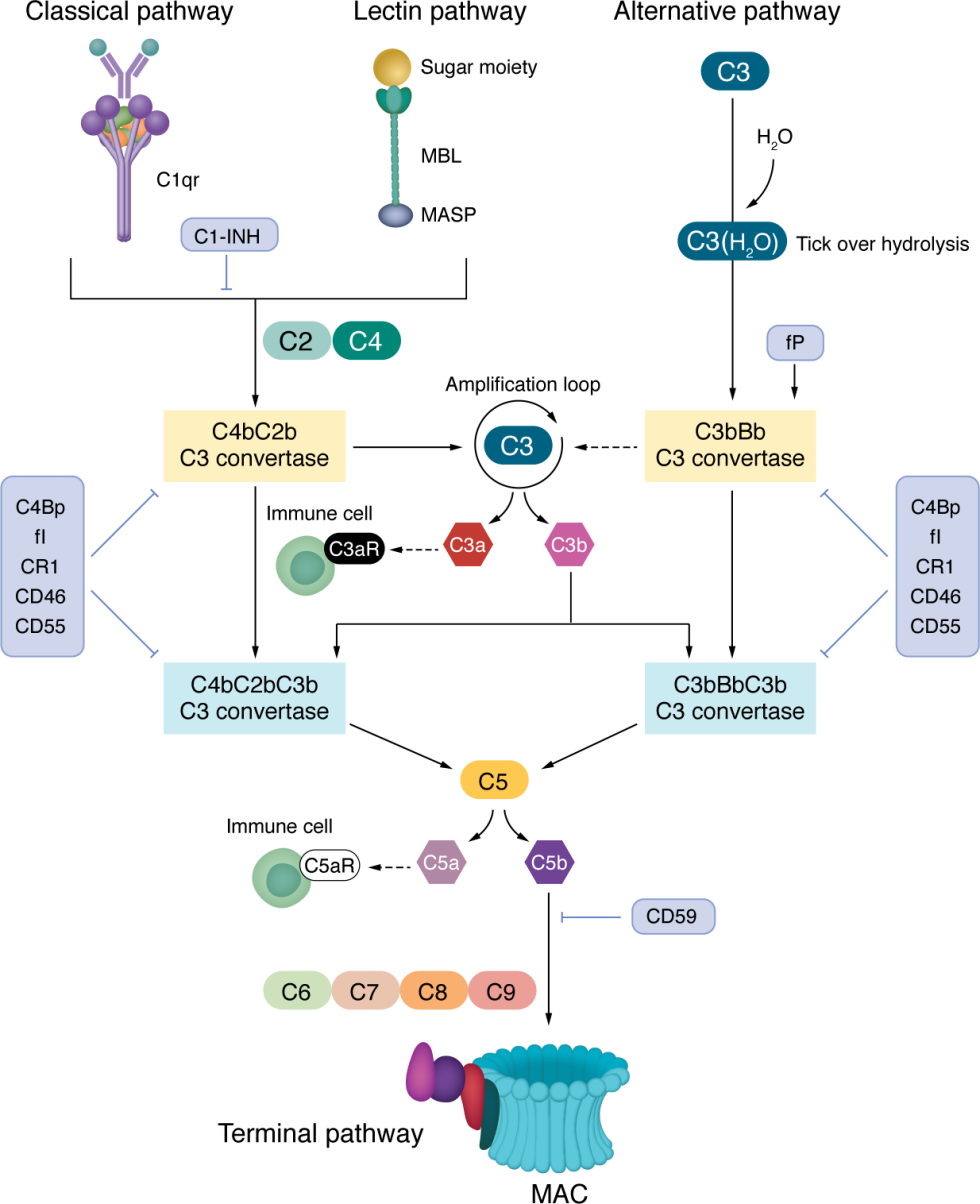
Complement activation and regulation. Binding of C1q to IgG or IgM antibody–opsonized pathogens or altered host cells initiates the classical pathway, whereas binding of MBL to sugar motifs on the surface of bacteria initiates the lectin pathway. The alternative pathway is initiated via spontaneous cleavage of the complement protein C3 into C3a and C3b. All three pathways converge onto the complement protein C3, which is cleaved into C3a and C3b by the C3 convertases (C4b2a and C3bCBb). The generation of C3 convertases amplifies the complement cascade through continued cleavage of C3. C3b opsonizes microbes and targets them for destruction by phagocytes. C3b combines with other complement proteins to generate the C5 convertases (C4b2a3b and C3bBb3b), which cleave C5 into C5a and C5b (14, 129). C3a and C5a are complement anaphylatoxins that bind to their respective cell surface receptors, C3aR and C5aR, and promote migration to and activation of immune cells at sites of infection or damage (130, 131). C5b binds to complement proteins C6, C7, C8, and C9 to generate the MAC, which forms a pore in the cell membrane and induces lysis of bacterial or complement-targeted host cells (11). CD55 is a membrane-bound complement regulator that disassembles C3 and C5 convertases to prevent amplification of the cascade and the generation of the MAC (12). CD46, also known as membrane cofactor protein (MCP), works with serum factor I to prevent C3 convertase reassembly and amplification of the cascade. CD46 functions are well characterized in humans but a functional mouse homolog has not yet been identified (132). CD59 is a complement regulator that blocks MAC formation. The complement regulator CR1, which exists in a soluble (sCR1) and membrane-bound form, acts as a receptor for C3b and C4b, thereby destabilizing and enhancing decay of the classical and alternative pathway C3 and C5 convertases (133).

The complement system represents one of the most ancient and conserved mechanisms of defense, which has been transmitted throughout the evolution from invertebrates to humans (5). Besides playing a key role in the defense against infections, an overwhelming number of studies have shown that the complement system is critical in maintaining health and homeostasis in the entire human body and that its functions can go well beyond providing defense against pathogens. As such, it is not surprising that dysregulation of the complement system has been described in the development and progression of diseases spanning broad etiologies and arising in multiple body sites, from autoimmunity to neuroinflammation and cancer, as reviewed elsewhere (6–9). The complement system comprises more than 50 proteins with major roles in the innate immune response against pathogens and altered host cells via opsonization, inflammation, and cell lysis. These proteins can be found in circulation, on the cell surface, or within cells and play critical roles in the initiation, amplification, effector function, or regulation of the complement cascade. In the complement cascade, a series of enzymatic cleavages are initiated upon the recognition of foreign or altered cells by various complement proteins (10). The cascade can be initiated via the classical, the lectin, or the alternative pathways. Although distinct, all three pathways lead to the generation of the C3 convertases, which mediate the cleavage of C3 into C3a and C3b and allow the formation of the C5 convertase. This step is essential to produce the downstream proteins that form the membrane attack complex (MAC) (11). To prevent aberrant activation, the complement system possesses distinct regulatory mechanisms that target specific steps within the cascade (12–14) (Figure 2).

Traditionally, cleavage of C3 into its soluble and membrane-bound fragments was believed to be confined to the extracellular space both for systemic and cell-derived complement. However, seminal work by Kemper and collaborators revealed the existence of an intracellular complement system, termed the complosome, whereby the production and activation of the cascade remain confined within cells (15). These studies shed light on novel noncanonical functions of the complement system, sparking exciting research that is fundamentally changing our understanding of complement and its roles in health and diseases. Besides multiple immune cell types, cell-autonomous complement has been described in endothelial and epithelial cells in intestine, lung, bladder, kidney, and eye as well as in pancreatic β cells, hepatocytes, fibroblasts, and neurons, orchestrating responses to infectious and noninfectious stimuli (16–21). More extensive reviews on the functions of intracellular complement have been previously published (6, 8, 22). However, evidence for a role of the intracellular complement in intestinal inflammation and colon cancer is still limited, so here we focus primarily on extracellular complement signaling.

**Figure 3:**
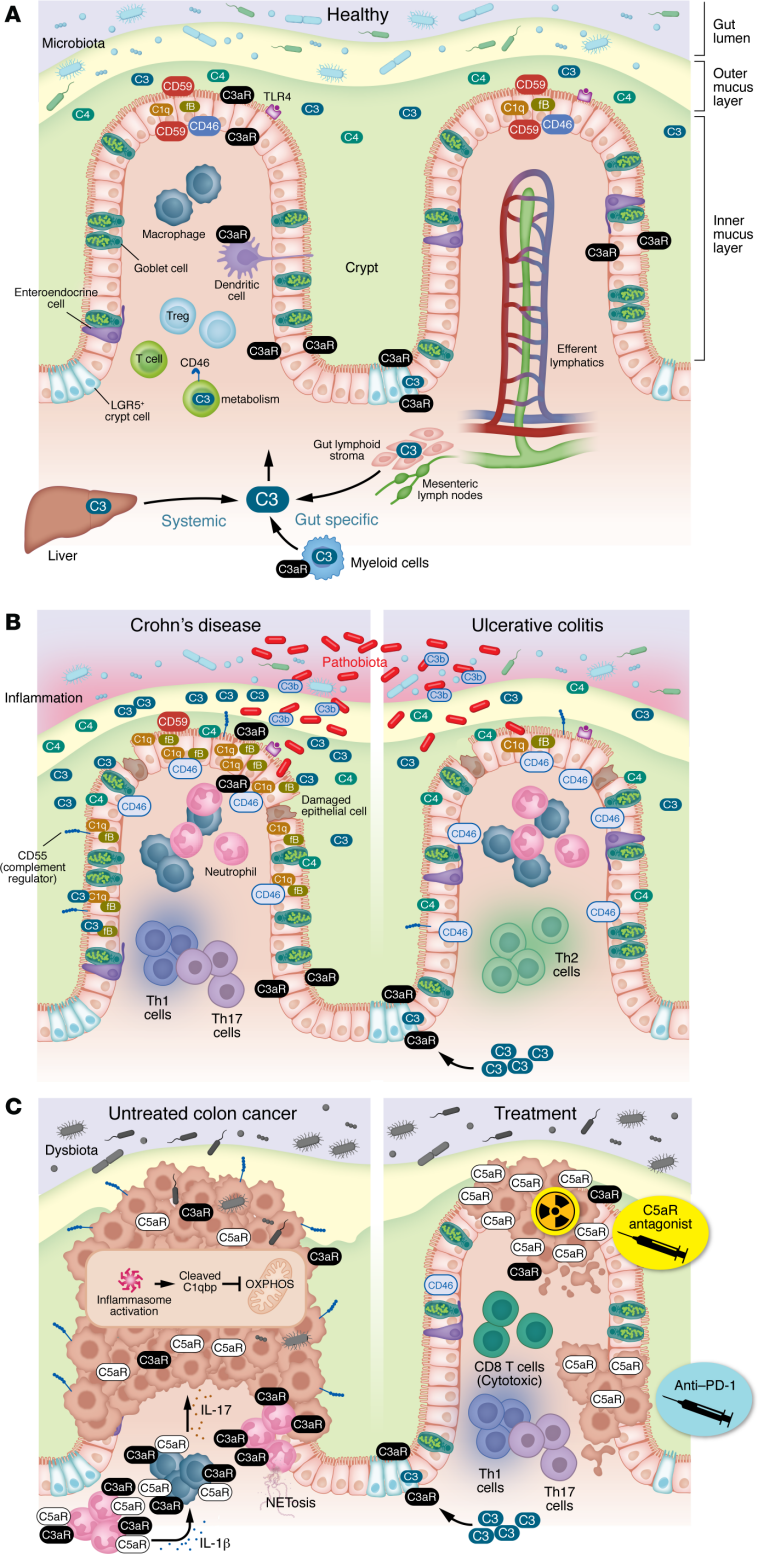
Complement in healthy colon, IBD, and colon cancer. (**A**) In healthy colon local C3 production is dependent on the commensal flora, and stromal and myeloid cells are the major producers (23). IECs constitutively produce and secrete C3 and express the C3aR and TLR1–4. C3a and its degradation product C3a-DesArg have potent antibacterial and antifungal properties independent of their interaction with C3aR (134). Primary IECs do not express C5aR, indicating that C5aR expression may be more prominent during intestinal inflammation (135, 136). Lgr5^+^ stem cells in the SI express C3aR, which acts as an autocrine growth factor necessary for the proliferation of the intestinal organoids (137). C3aR is mostly expressed in lamina propria eosinophils, CD11b^+^ and CD103^+^ conventional dendritic cells, plasmacytoid dendritic cells, and a fraction of innate lymphoid cells (138). Tregs, macrophages, and dendritic cells support intestinal homeostasis. (**B**) In inflammatory bowel disease, the reduced mucus layer enables proliferation of pathobionts and their proximity to the intestinal epithelium, causing migration of innate and adaptive immune cells, inflammation and disruption of the intestinal barrier. C3 activation leads to opsonization of mucosa-associated bacteria by C3b. Crohn’s disease involves Th1/Th17 inflammation and higher levels of C3, C1q, and CfB. Ulcerative colitis results in Th2 inflammation and no induction of C3. (**C**) Alterations of the mucus layers and microbiota occur in colon cancer. Untreated tumors express CD55 and C5aR. Inflammasome activation results in cleavage of C1qbp, preventing its translocation to the mitochondria, inhibiting OXPHOS, and supporting cancer cell proliferation. C5aR-expressing neutrophils produce IL-1β, which stimulates macrophage IL-17 production. C3aR on neutrophils induces NETosis. Both mechanisms support tumor growth. Radiation therapy induces C5aR overexpression on cancer cells. Treatment with a C5aR antagonist causes tumor cell death and improved response to radiotherapy. Loss of C3aR results in increased recruitment of Th1, Th17, and cytotoxic CD8 cells and improved response to anti–PD-1 therapy.

As an essential component of host immunity, the complement system is gaining increasing attention in the gastrointestinal (GI) tract, where its functions have been described in intestinal homeostasis, inflammation, and cancer. In this context, a recent study by Wu and collaborators provided compelling evidence for the existence of a gut-intrinsic complement system that acts independently from liver-derived complement, further validating the concept of tissue-specific complement already described in lung and brain (23). In the intestine, the complement system cooperates with other innate sensing mechanisms. Multiple studies have provided evidence for crosstalk between the complement system and the TLRs (24–26). More recently, Kopp et al. speculated that there may be crosstalk between complement and Paneth cells, whereby complement proteins such as the anaphylatoxins C3a and C5a and/or the MAC could enhance or dampen antimicrobial peptide (AMP) production or whereby AMPs drive the intracellular production of C3 and C5 by specialized IECs (27). This hypothesis is supported by a study by Cheoub et al. showing that C5aR antagonism resulted in a decline in diversity of the skin microbiota of healthy mice associated with changes in immune effectors, including AMPs. Besides its crosstalk with other innate sensing mechanisms, multiple components of the complement system regulate the function of IECs and immune cells, thus contributing to intestinal homeostasis in the healthy intestine (Figure 3A).

## Role of the complement system in intestinal inflammation

As the largest mucosal surface of the human body, the GI tract is constantly exposed to environmental stimuli, mainly via the introduction of new antigens derived from food, toxins, and the luminal flora. The highly compartmentalized structure of the intestine allows selective absorption of nutrients and water while selectively limiting permeation of luminal toxins, antigens, and pathobionts through the mucosa, ultimately preventing excessive inflammation. Disruption of this delicate balance can result in intestinal inflammation and in the development of IBD, which has been the subject of multiple investigations in the complement field. Crohn’s disease (CD) and ulcerative colitis (UC) are the two main forms of IBD, and they differ in terms of location, histopathology, and immune responses, as extensively reviewed by others (28, 29). Since the complement system plays key roles in innate defense and in the regulation of various cell functions, it is not surprising that many investigations have focused on studying its relevance in the context of IBD. These studies are summarized in Table 1. An overview of the involvement of complement components in IBD is provided in Figure 3B.

### Complement system in patients with IBD.

The first evidence for complement involvement in IBD came from studies showing increased levels of complement factor B (CfB) and C3 in the sera of patients with IBD and their correlation with disease activity (30, 31). Additional evidence of complement activation at the protein level in patients with IBD came from a study by Ueki and collaborators, which investigated the expression of C3 activation and degradation products, terminal complement complex (TCC), and complement regulatory proteins in biopsies from patients with IBD. Interestingly, this study also showed that CD55 was mainly expressed on the apical membrane of the epithelium, while CD46 was mainly found in the basolateral membrane (32). These findings contrast with those from studies showing that C3 and TCC were expressed on the apical membrane of IECs in patients with IBD and that CD59 was specifically reduced on IECs but not other cell types in patients with UC and CD, potentially causing complement-mediated destruction of the intestinal epithelium (33–35). These discrepancies may be explained by differences in antibody specificity, methods for tissue preparation, imaging techniques, and patients’ pathology. Regarding the latter explanation, Halstensen et al. showed that patients with CD exhibited significant deposition of C3b in the mucus layers, while patients with UC expressed IgG1 and C4c in addition to C3b, leading to the hypothesis that the classical pathway of complement activation may be more relevant in UC than CD (36). In agreement with these findings, Sünderhauf and collaborators found that C3 expression at the mRNA level was upregulated exclusively in colonic biopsies of patients with CD who were in remission and had no signs of inflammation compared with colon biopsies from healthy individuals and patients with UC (26). Interestingly, in colons from patients with CD with active inflammation, levels of C3 were comparable to those of their noninflamed counterparts but significantly higher than those of patients with UC and patients acting as controls, and there was a significant upregulation of CfB (Figure 3B). Notably, C5 levels were undetectable in the colonic biopsies, and although this study did not specifically assess protein expression level, this result confirms previous findings that argued against terminal activation of the complement pathway in IBD (32). Additional studies examining the potential differences in complement activation between patients with UC and CD found that patients with CD exhibited enhanced levels of C1q and C3 at the mRNA and protein levels and an increased number of IgM^+^ B cells compared with patients with UC irrespective of the inflammatory status (37).

Interestingly, Laufer and collaborators showed that C3 was limited to inflamed tissues, where its expression was observed in IECs and in a population of CD68^+^ macrophages that formed an inflammatory infiltrate in the crypts (38). In contrast, C4 mRNA was expressed diffusely by enterocytes both in patients with CD and in healthy individuals acting as controls and was also found in the mast cells in the submucosa of patients with CD, providing further evidence for the role of these cells as an additional source of complement in the intestine that may be relevant in health and disease (39). Higher C4 levels were also described by others in the jejunal fluids of patients with CD, where activated intestinal monocytes and macrophages were hypothesized to be the most likely source (40). Intriguingly, upregulation of the complement regulator CD55 was observed in patients with IBD, which may indicate that some components generated during the complement activation cascade may exert protective functions in intestinal inflammation (Figure 3B). However, based on the observation that CD55^–/–^ mice were more susceptible to chemically induced colitis, the authors concluded that upregulation of CD55 may represent an attempt to block excessive complement activation (41).

There is also evidence for the involvement of the complement system in the regulation of the microbiome, which is highly relevant to IBD etiology. Specifically, Nissila et al. found that in pediatric patients with IBD the levels of *C4B* positively correlated with inflammation, and patients lacking *C4B* had enhanced microbiota diversity compared with patients with two copies of *C4B*. These findings point toward a potential role of C4B in exacerbating IBD-related dysbiosis via promoting excessive complement reactivity toward the intestinal flora (42).

### Complement system in experimental models of intestinal inflammation.

The evidence of complement involvement in IBD pathology in patients fueled multiple studies in animal models with the aim of dissecting the underlying mechanisms and identifying potential therapeutic avenues.

Sünderhauf et al. found that mice with active dextran sulphate sodium–induced (DSS-induced) colitis underwent significant transcriptional upregulation of *C3*, but not other complement components such as *C2, Cfb, C5ar1, C5ar2,* and *C3ar*, in the colon and in primary IECs. Interestingly, this study also reported that C3b fragments adhered to mucosa-associated bacteria, potentially favoring their phagocytosis by myeloid cells (26). These findings further suggest that complement activation may be secondary to disruption of the mucus layer that brings bacteria and epithelial cells into closer proximity, ultimately causing an inflammatory immune response.

Other studies found C3aR and C5aR upregulation in the inflamed tissues of DSS-treated mice, which led to more thorough investigations of their role in acute and chronic intestinal inflammation. Using full-body knockout mice, Wende and colleagues showed that C3aR was mildly detrimental during acute DSS-induced colitis in Th2-biased BALB/c but not in Th1-biased B6 strains of mice (43). In the absence of C3aR, BALB/c and B6 mice developed differing cytokine profiles during DSS-induced colitis, consistent with the described differences in T cell polarization between these two strains (43–45). These strain-specific findings are complicated to translate to human IBD. The same group also studied the effect of C5aR on intestinal inflammation, demonstrating that global lack of C5aR was protective against acute DSS-induced colitis (46). These findings are in agreement with reports showing that the use of C5aR antagonists is protective in acute models of colitis induced by DSS or by 2,4,6-trinitrobenzene sulfonic acid administration, which mimics the pathology of CD (47–49). Interestingly, in a model of chronic colitis, whereby multiple cycles of DSS administration are followed by recovery to more closely mirror the relapse/remission episodes observed in patients, Johswich and collaborators found that C5aR deficiency was detrimental and resulted in greater weight loss and intestinal pathology. During chronic inflammation, C5aR^–/–^ mice exhibited increased IL-5 and reduced IL-4 and IFN-γ transcripts compared with WT mice (46). These differences may be at least in part due to the microbiome, which was not specifically examined in this context, or by the different roles that C5aR plays in multiple cell types depending on its subcellular locations (50–52). Furthermore, the study by Johswich also found that during chronic inflammation, C5aR^–/–^ mice underwent a significant reduction in the levels of C3aR, despite these mice having an even higher number of myeloperoxidase-expressing neutrophils than in WT animals. This observation is particularly intriguing, as it suggests a potential direct or indirect role of C5aR on the expression of C3aR, which could exert a protective effect during chronic intestinal inflammation (46). Additionally, it is possible that, while C5aR deficiency protects from tissue damage during active disease, it is detrimental during the remission phase owing to its role in repair/regeneration. Accordingly, another study showed that in chronic colitis levels of C5aR were reduced in the colon during active inflammation but increased in the remission phase (53).

In line with the hypothesis that certain complement proteins may be protective against intestinal inflammation, C3 deficiency in mice was shown to promote the loss of E-cadherin, tight junctions, and ion channels and to increase inflammatory responses via the inducible nitric oxide synthase–mediated cyclooxygenase-2 pathway, with upregulation of TNF-α, IL-1 and IL-6 (54). Furthermore, C5^–/–^, C1q^–/–^, MBL^–/–^, C3^–/–^, fB^–/–^, and C6^–/–^ mice all show increased susceptibility to acute and chronic DSS-induced colitis, further highlighting the important role of the complement system in the regulation of intestinal immune responses (53, 55–57). In the same studies, treatment with complement inhibitors (CR2-fH) targeted to sites of complement activation or inhibiting the alternative pathway effectively prevented excessive intestinal inflammation, reduced fibrosis, and lowered the number of infiltrating macrophages and B cells (53).

Overall, it remains unclear whether the complement system is a passenger or a driver in IBD. In a study aimed to characterize the contribution of rare genetic variants to IBD development, Shaw and collaborators found that mutations in complement *C2*, *C3*, and *CFB* were among the top 200 most significant common variants associated with disease in a cohort of 628 pediatric patients (58). Multiple mouse models lacking specific complement factors do not spontaneously develop intestinal inflammation, suggesting that mutations in complement genes may not be sufficient to drive IBD and that the condition requires a “second hit.” This is particularly intriguing considering that similar findings have been shown for mutations in other genes implicated in innate immunity and host microbiome interaction that are strongly associated with IBD. For instance, the great majority of individuals homozygous or heterozygous for NOD2 variants as well as *Nod2*-deficient mice housed under specific pathogen–free conditions do not develop CD without the presence of additional genetic hits or specific pathobionts (59–61).

## Complement in colon cancer development, progression, and treatment

Activation of the complement system and its ability to tag, clear, lyse, and stimulate antibody-mediated cytotoxicity against foreign or altered cells has traditionally been seen an important aspect of immunosurveillance (62). As such, activation of the complement system has been described in multiple cancer types in human and preclinical models (63–65) (Table 2). However, owing to its ability to promote inflammation, complement can also play an active role in the development and progression of multiple cancers (66). Cancer cells not only have the ability to express higher levels of complement regulatory factors to evade complement-mediated killing, but in many instances they can produce and activate complement themselves, which suggests that complement is a double-edged sword in cancer and its roles go well beyond traditional immunosurveillance (67–70).

### Complement system functions in colon cancer cells.

In the context of colon cancer, there have been important insights from studies investigating the role of the complement system in the intestine using various colon carcinoma cell lines as surrogate of IECs. Andoh and collaborators reported that Caco-2 cells derived from human colon adenocarcinoma were able to generate functional C3, C4, and CfB and that the production of these complement factors could be further enhanced by stimulation with cytokines such as IL-1, TNF-α, IL-6, and IFN-γ (71). However, the functional role of complement production in cancer cells was not investigated. Other studies have shown that Caco-2, T84, and HT29 colon cancer cell lines can also respond to the complement split products C3a and C5a via expression of complement anaphylatoxin receptors on their apical membrane. Stimulation of these cell lines with C3a or C5a led to increased mRNA levels of CXCL2, CXCL8, CXCL11, CXCL10, and IL-8 following activation of the ERK pathway, which resulted in increased monolayer permeability (72, 73). However, the functional relevance of increased intestinal permeability is questionable in the context of colon cancer, where disruption of the epithelial barrier has already occurred. Moreover, other studies have shown that ERK activation is essential for the normal expression of key tight junction proteins and regulation of epithelial integrity (74, 75). Perhaps more functionally relevant in the context of cancer is the finding that C5a-induced IL-8 production enhanced the proliferation of colon cancer cell lines (73).

Additional findings support the role of complement in regulating cancer cell metabolism. C1Q binding protein (C1QBP) has been consistently found to be upregulated in multiple cancers (76–78). In patients with colon cancer, high C1QBP expression correlated with worse overall survival (OS) compared with patients with low C1QBP, and it was shown to interact with apolipoprotein A-I, potentially explaining the well-established antitumor effects of this high-density lipoprotein in human and preclinical models (79–81). Interestingly, Sünderhauf and collaborators found that C1QBP cleavage by caspase-1 prevented its import to the mitochondria, which resulted in reduced oxidative phosphorylation activity and enhanced glycolysis in HT29 colon cancer cell lines, ultimately increasing proliferation (Figure 3C). Although caspase-1 and C1QBP levels did not correlate with tumor stage, they were increased in human colon cancer tissues compared with the paired normal mucosa, demonstrating the potential relevance of inflammasome and complement interaction in patients with colon cancer (82).

### Investigation of complement in human colon cancer.

An analysis of The Cancer Genome Atlas datasets revealed that complement mutations occurred at high frequencies in multiple cancers and were associated with a robust reduction of OS in patients with colon cancer. Furthermore, the authors demonstrated that hypoxia supports the upregulation of CD55 on colon cancer cell lines, which results in protection from complement-mediated cytotoxicity, thus unraveling a novel crosstalk between complement and hypoxia. Interestingly, cancer-associated mutations resulting in higher CD55 expression correlated with worse disease-free survival (83). Prostaglandin E2 (PGE2), which is well known to promote colon cancer, was also shown to induce CD55 expression in cancer cells and in spontaneous models of intestinal tumorigenesis. Inhibition of CD55 was effective at restraining colon cancer development and metastases, further supporting the tumor-promoting role of this complement regulator (84–86) (Figure 3C).

Multiple studies found that certain complement mutations are more prevalent in specific ethnicities. For instance, loss-of-function mutations in complement *C2* have been described to be more prevalent in populations with European ancestry, while loss-of-function mutations in complement *C9* are more prevalent among those with Japanese ancestry (87, 88). Mutations in complement *C5* and *C6* appear to be highly prevalent in populations with African ethnicity in sub-Saharan Africa and among African American populations of the Southeastern region of the US, respectively (89, 90). In the case of C5, the single nucleotide polymorphism c.754G>A:p.A252T, which is common in the Western Cape population, is found in 7% of the African population with meningococcal disease. Despite affecting a small part of the full-length C5, the hypothesis is that this mutation may lead to longer folding times, ultimately affecting the C5 intracellular stability or secretion (89, 91). While it is well known that *C5* and *C6* mutations predispose to 10,000-fold higher frequencies of *Neisseria* *meningococcal* infections in these populations, their effect on colon cancer development, progression, and response to treatment remains largely understudied (92–94). This is a potentially interesting area of investigation, especially considering that populations of African ancestry are often diagnosed with more aggressive forms of colon cancer, which overall result in worse outcomes compared with populations of European ancestry (95, 96).

Mutations causing deficiencies in the lectin pathway of complement activation were initially hypothesized to predispose to increased risk of developing postoperative infections in patients with colon cancer, ultimately resulting in higher recurrence rates and mortality. However, studies by Ytting and collaborators showed that while the frequency of mannose binding lectin (MBL) mutations between healthy individuals and patients with colon cancer did not differ, the serum levels of MBL, MASP2 and the activity of MBL/MASP were significantly increased in the blood of patients with colon cancer, and high MASP-2 levels in serum proved to be an independent prognostic marker to predict recurrence and poor survival (97, 98).

Recognition of alteration of the complement system in human colon cancer also emerged from the first molecular characterization of colon cancer subtypes (99). That study identified four main colon cancer molecular subtypes (CMS1–4), with CMS1 cancers characterized by a distinct immune signature typical of microsatellite instability high (MSI-H) cancers, CMS2 cancers with *MYC* and *WNT* activation, CMS3 cancers with metabolic deregulation, and CMS4 tumors with a mesenchymal phenotype characterized by upregulation of TGF-β, angiogenic factors, and stromal infiltration. Interestingly, CMS4 cancers showed a distinct upregulation of the complement pathway. Although the complement genes responsible for driving the upregulation of this pathway in the CMS4 cancers were not specifically investigated in this study, the findings that patients with these cancers had worse OS and relapse-free survival compared with patients with other molecular subtypes suggest a detrimental role of the complement system in colon cancer prognosis (99). On the other hand, there is evidence that C3 is significantly downregulated in paired cancer and healthy colon tissue from patients with cancer compared with colon tissue from patients without cancer (100). Although this latter study did not assess the correlation between C3 expression and survival, the results suggest that the loss of C3 may favor tumor development. An analysis of publicly available datasets indicated that C3 expression was higher at the gene and protein level in patients with colon versus rectal cancer. Intriguingly, C3 expression in patients with colon cancer was strongly associated with antigen processing and presentation, neutrophil effector functions, B cell maturation, and humoral immune response pathways (101). Furthermore, C3 expression was associated with increased immunoscore, which is a positive prognostic factor in colon cancer and confers susceptibility to immune checkpoint blockade immunotherapy (ICI) (102–104). While the relationship between C3 expression and response was not investigated, in the above-mentioned study, C3 expression correlated with worse OS. These finding may appear contradictory. However, they are reminiscent of a trend previously described in the MSI-H colon cancers, in which the immune infiltrate is a prognostic factor associated with significantly better survival than microsatellite stable (MSS) colon cancers in the early stages but with worse survival in the metastatic setting (103, 105).

### Complement anaphylatoxins in preclinical models of colon cancer.

Complement anaphylatoxins have been extensively investigated in preclinical models of colorectal cancer because of their known role in promoting inflammation and recruitment of immunosuppressive cells of myeloid origin in the context of other malignancies. In a model of inflammation-induced colon cancer, Ning and collaborators observed reduced tumor development in the colons of C3^–/–^ mice, suggesting a protumorigenic role of the complement system. Mechanistically, they showed that C5a release during tumor development stimulated the production of IL-1 by neutrophils, which acted in an autocrine and paracrine manner on mucosal myeloid cells and induced production of IL-17A, ultimately activating the STAT3/NF-κB–dependent pathway responsible for the hyperproliferation of the cancer cells (106) (Figure 3C). Piao et al. further showed that the C5a/C5aR axis promotes metastases formation in an experimental model of colorectal liver metastases by stimulating in the macrophages the production of monocyte chemoattractant protein (MCP-1) via the AKT signaling pathway. This resulted in increased recruitment of macrophages, neutrophils, and dendritic cells to the metastatic niche and promoted production of arginase, IL-10 and TGF-β, contributing to the development of a protumorigenic microenvironment and supporting the growth of metastases. In line with the findings by Ning et al., Piao et al. showed that C5aR expressed by the immune cells was mainly responsible for the observed effects. Additionally, while C5aR ablation significantly reduced the metastatic burden, it did not completely abrogate metastases, indicating that other mechanisms in addition to the C5a/C5aR axis are required for the establishment of colorectal liver metastases (107). Interestingly, in both studies, bone marrow chimera experiments demonstrated that only the complement expression in immune cells conferred a protumorigenic phenotype and promoted the formation of metastases. However, other studies have shown that cancer cell–intrinsic C5a/C5aR signaling may also play a role in intestinal tumorigenesis. Specifically, intracellular C5a stimulation of C5aR1 causes β-catenin stabilization and promotion of colon cancer (108). Along the same line, a study by Olcina and collaborators demonstrated that the C5a/C5aR axis in colon cancer cells plays a key role in their response to treatment. Specifically, by transplanting tumoroids that closely resembled the patient tumor microenvironment (TME), the authors found that C5aR1 expression was induced in the cancer cell as part of the stress response to radiotherapy. Inhibition of C5aR1 resulted in increased NF-κB–dependent apoptosis specifically in tumors, but not in normal tissues, and significantly improved the response to radiotherapy even in tumors with immunosuppressive features (Figure 3C). Notably, these effects were likely independent of T cells, as the reduced tumor growth was observed also in athymic mice, suggesting that C5aR1 inhibition plus radiotherapy may be an efficient combination treatment, even in tumors with immunosuppressive features and low T cell infiltration, which constitute most colon cancer cases (109).

Another study identified the crosstalk between dietary fats and the complement system as an important promoter of intestinal neoplasia. In this study, the authors took advantage of the well-established model APC^Min/+^ mice, which spontaneously develop polyps along in the intestine, as well as multiple mouse models with enhanced susceptibility to high-fat diet (HFD) treatment. Mechanistically, they showed that specific dietary fats such as hydrogenated coconut and corn oil but not olive oil activated the complement system, leading to the generation of C5a and upregulation of the proinflammatory cytokines MCP-1, IL-1β, IL-6, IL-23, TNF-α, and VEGF in adipose tissue, intestine, and blood, which correlated with increased tumorigenesis. Importantly, lean mice fed HFD presented increased inflammation and tumorigenesis, indicating that obesity and metabolic status were not the drivers of C5a-induced tumorigenesis. Pharmacologic and genetic targeting of C5aR prevented diet-induced local and systemic inflammation and significantly reduced polyp burden (110). Since the specific source of complement was not investigated in this study, it is not clear whether immune or nonimmune cells contributed to its production in response to HFD.

Using APC^Min/+^ mice, our group found that during spontaneous intestinal tumorigenesis there was an increase in protumorigenic low-density neutrophils with the ability to spontaneously release neutrophil extracellular traps (NETs). We showed that LPS, which was increased in the circulation of tumor-bearing mice as result of intestinal barrier dysfunction, induced activation of the complement cascade via the alternative pathway. These findings are in line with those of a previous study showing that the alternative pathway of complement activation was increased in patients with cancer, and this increase remained unaltered after surgery (111). Interestingly, LPS also induced upregulation of C3aR on neutrophils, which led to NETosis-dependent hypercoagulation and N2-phenotype (or tumor-promoting neutrophil) polarization, ultimately fueling tumorigenesis (Figure 3C). Ablation of C3aR in APC^Min/+^ mice (APC^Min/+^C3aR^–/–^) normalized the neutrophil counts, resolved the hypercoagulation, and significantly reduced tumor burden and NETs, demonstrating the relevance of this novel crosstalk between complement, neutrophils, and coagulation in intestinal tumorigenesis (112). Despite undergoing a significant reduction in small intestinal tumors, APC^Min/+^C3aR^–/–^ mice showed a striking increase in number of colon tumors, which, although surprising, may be explained by the known differences between large and small intestine and by the potentially different functions of the C3a/C3aR axis in these specific compartments. These findings were not limited to the genetic model but could be recapitulated in an inflammation-driven model of colorectal cancer. Furthermore, we described increased permeability of the gut vascular barrier in APC^Min/+^C3aR^–/–^ mice, which facilitated the translocation of bacteria to the liver to promote a premetastatic niche and favored the hematogenous dissemination of colon cancer cells with consequent development of metastases (113). By performing fecal microbiota transplantation, we proved that C3aR deficiency results in the development of a protumorigenic microbiota characterized by an increase in Bacteroidota (previously Bacteroidetes) and Gammaproteobacteria phyla. Furthermore, we showed high degree of immune infiltration in the colon tumors from C3aR^–/–^ mice dominated by Th1, Th17, and cytotoxic T cell signatures. This immune signature conferred susceptibility to ICI and effectively reduced tumor growth in otherwise ICI-unresponsive APC^Min/+^ tumors.

By mining publicly available datasets, we found that C3aR downregulation occurs in subsets of human colon and rectal cancers independently of their MSI-H or MSS status and is driven by *C3AR1* gene methylation. In line with our findings in the mouse models, in patients, reduced levels of C3aR expression correlated with increased accumulation of innate and adaptive immune responses in the TME, which supports antitumor immunity (114) (Figure 3C). It is interesting to note that, while during tumor development C3aR has a protective effect, in therapeutic settings the ablation of C3aR achieved comparable results to ablation of C5aR described in the context of ICI treatment in other cancers (115, 116).

Based on these findings, it is tempting to speculate that the C3a/C3aR axis regulates key physiologic functions of the large intestinal epithelium and, as such, loss or downregulation of C3aR could represent the Achilles’ heel of colon cancer. The complement system at mucosal surfaces such as the GI tract is essential to avoid overt inflammation by keeping immune responses to microbial flora in check. However, during tumor development and therapy, this regulatory mechanism may restrain the activation of immune responses and the C3a/C3aR axis can act as an immune checkpoint that prevents antitumor immunity in colon cancer.

## Summary and future directions

A growing number of studies are contributing to deciphering the canonical and noncanonical functions of the complement system in the GI tract in homeostatic conditions, during inflammation, and in tumor development, progression, and treatment. The normal intestinal epithelium can produce its own complement factors, which can act in an autocrine or paracrine manner. Similarly, cancer cells can also produce and respond to complement factors and express complement inhibitory molecules. Overall, during intestinal inflammation and tumorigenesis the complement system can exert both protective and detrimental roles. The apparent contradictory results emerging from both human and preclinical studies may be explained by the distinct roles that the complement system plays not only in different cancers but also in different phases of the same cancer. While complement may participate in tumor destruction, it can also be exploited by cancer and immune cells to ultimately support tumor growth via different mechanisms. These paradoxical functions position the complement system within the framework of the 3C and 3E theory of immunoediting in cancer (117–119).

The discovery of the complosome, the identification of novel noncanonical functions of the complement proteins, and the availability of novel genetic models to dissect the function of specific complement factors in a cell-specific manner now make it possible to further explore this hypothesis and may lead to exciting developments in our understanding of role of the complement system in the GI tract.

## Figures and Tables

**Table 2:**
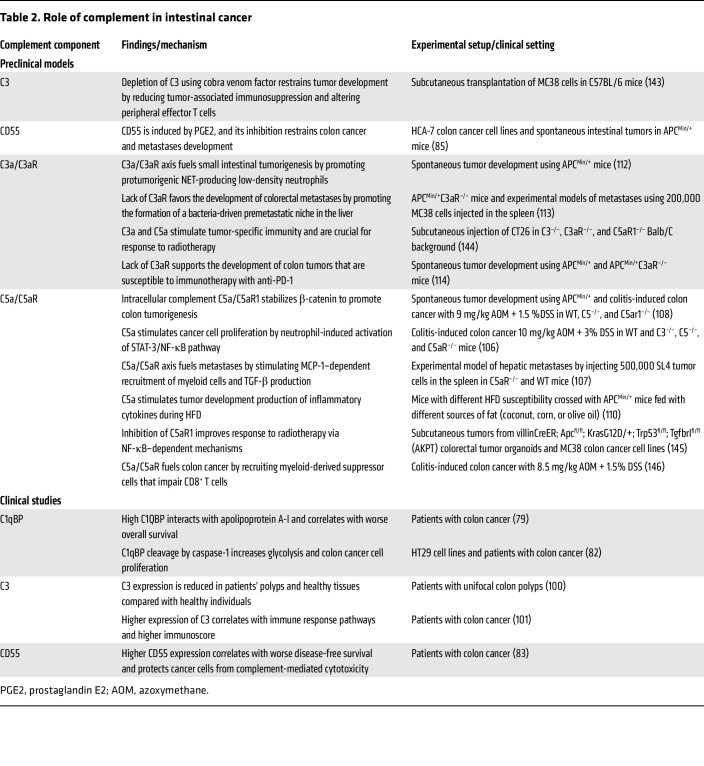
Role of complement in intestinal cancer

**Table 1:**
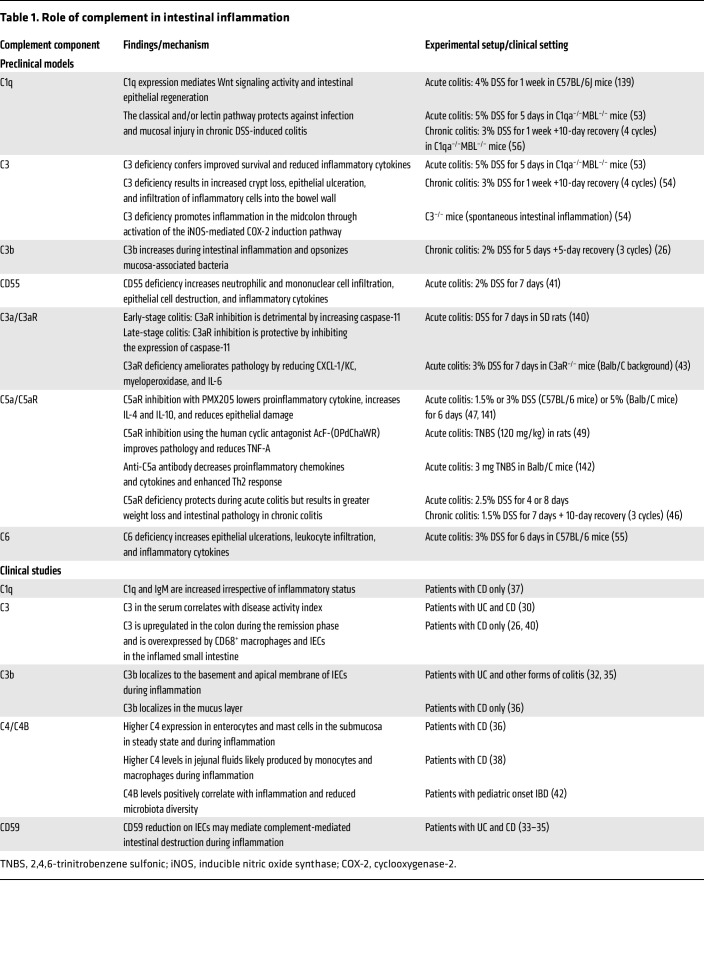
Role of complement in intestinal inflammation

## Version 1 10/01/2025

Electronic publication
